# High Level Expression and Purification of Cecropin-like Antimicrobial Peptides in *Escherichia coli*

**DOI:** 10.3390/biomedicines10061351

**Published:** 2022-06-08

**Authors:** Chih-Lung Wu, Ya-Han Chih, Hsin-Ying Hsieh, Kuang-Li Peng, Yi-Zong Lee, Bak-Sau Yip, Shih-Che Sue, Jya-Wei Cheng

**Affiliations:** 1 Department of Medical Science, Institute of Biotechnology, National Tsing Hua University, Hsinchu 300, Taiwan; s103080578@m103.nthu.edu.tw (C.-L.W.); s9980517@m99.nthu.edu.tw (Y.-H.C.); s109080583@m109.nthu.edu.tw (H.-Y.H.); richard850210@gapp.nthu.edu.tw (K.-L.P.); g15004@hch.gov.tw (B.-S.Y.); 2 Institute of Bioinformatics and Structural Biology, National Tsing Hua University, Hsinchu 300, Taiwan; yzlee@gapp.nthu.edu.tw; 3 Department of Neurology, National Taiwan University Hospital Hsinchu Branch, Hsinchu 300, Taiwan

**Keywords:** antimicrobial peptide, expression, intein, self-cleavage, cecropin-like

## Abstract

Cecropins are a family of antimicrobial peptides (AMPs) that are widely found in the innate immune system of Cecropia moths. Cecropins exhibit a broad spectrum of antimicrobial and anticancer activities. The structures of Cecropins are composed of 34–39 amino acids with an N-terminal amphipathic α-helix, an AGP hinge and a hydrophobic C-terminal α-helix. KR12AGPWR6 was designed based on the Cecropin-like structural feature. In addition to its antimicrobial activities, KR12AGPWR6 also possesses enhanced salt resistance, antiendotoxin and anticancer properties. Herein, we have developed a strategy to produce recombinant KR12AGPWR6 through a salt-sensitive, pH and temperature dependent intein self-cleavage system. The His6-Intein-KR12AGPWR6 was expressed by *E. coli* and KR12AGPWR6 was released by the self-cleavage of intein under optimized ionic strength, pH and temperature conditions. The molecular weight and structural feature of the recombinant KR12AGPWR6 was determined by MALDI-TOF mass, CD, and NMR spectroscopy. The recombinant KR12AGPWR6 exhibited similar antimicrobial activities compared to the chemically synthesized KR12AGPWR6. Our results provide a potential strategy to obtain large quantities of AMPs and this method is feasible and easy to scale up for commercial production.

## 1. Introduction

Antimicrobial peptides (AMPs) normally consist of 12 to 50 amino acids and can be classified as α-helices [1], β-sheets [2], extended [3], and looped peptides [4,5]. Most AMPs exert their antimicrobial activities through the incorporation and permeabilization of microbial membranes, hence the death of microbial cells [6,7,8]. AMPs can work alone or in combination with antibiotics to diminish antibiotic-resistant pathogens and reduce the amount of antibiotics that are needed [9,10]. Recent progress of AMPs conjugated with antibiotics also demonstrated an enhanced killing effect on drug-resistant bacterial strains [11,12]. Moreover, many AMPs possess lipopolysaccharide (LPS) neutralization as well as anticancer activities [13,14,15]. Recent studies also summarized the immunomodulatory activities of AMPs in medical uses [15].

Cecropins are a family of AMPs that are widely found in the innate immune systems of Cecropia moths and are composed of 34 to 39 amino acids [16]. The structure of cecropins includes an N-terminal amphipathic α-helix, a hinge motif, and a hydrophobic C-terminal α-helix. This unique structural feature causes Cecropins or Cecropin-like peptides to possess various biological functions, such as antimicrobial activities [17], anti-inflammatory activities [18,19], and anticancer activities [16]. Previously, we developed a strategy to develop AMPs using a Cecropin-like hydrophilic helix—AGP-hydrophobic helix structural feature [20]. One of the leading peptides, KR12AGPWR6 (Ac-KRIVQRIKDFLR-AGP-RRWWRW-NH_2_) has been found to display superior antimicrobial activities, salt resistance, and LPS neutralizing activities [20].

There are two methods to produce AMPs, chemical synthesis and biosynthesis [21]. Chemical synthesis, such as solid-phase synthesis can provide efficiency and flexibility in the development of AMPs at a laboratory scale [22]. However, for AMPs with longer sequences (>25 amino acids), the yield and purity of chemical synthesized peptides will be reduced. Therefore, the cost of chemical synthesis has hindered the development of AMPs for industrial uses [23,24]. On the other hand, the biosynthesis of recombinant AMPs has several advantages, such as low cost, high yields, and a short production period. Among the expression systems, *Escherichia coli* is the most commonly used owing to its low cost of culture medium, high expression yield, and reduced production time [25]. The expression of AMPs has several challenges. Problems such as the toxicity of AMPs to host cells, their susceptibility to proteolytic degradation, and difficult purification procedures were found to be the major obstacles of biosynthesis. An effective strategy to overcome these limitations is to fuse the target AMP with a carrier protein and then release the peptide by enzymatic or chemical cleavage [24,26]. However, enzymatic cleavages such as thrombin [27], factor Xa [28], and enterokinase [29] were less efficient than chemical agents. On the other hand, chemical cleavage by CNBr or hydroxylamine has disadvantages, such as hazardousness and side-chain modifications [30,31,32]. Recently, we developed a salt-sensitive and self-cleavage *Nostoc punctiforme* (*Npu*) DnaE intein expression system [21]. The self-cleavage can be controlled by ionic strength, pH, and temperature. This intein expression system has several advantages, including high efficiency for self-cleavage, a quick purification process, no proteases or chemical reagents required for peptide release, and no residue in the final product [33,34,35]. Herein, we used this intein self-cleavage system to express and purify KR12AGPWR6. The antimicrobial activity of the biosynthesized KR12AGPWR6 was evaluated by MIC assay, and its structural feature was characterized by CD and NMR spectroscopy.

## 2. Materials and Methods

### 2.1. Chemicals and Reagents

The ECOS™ BL21 (DE3) competent Cells were purchased from Yeasterm Biotech Co., Ltd. (Taipei, Taiwan). Lysogeny broth (LB) was purchased from Cyrusbioscience (New Taipei City, Taiwan). Isopropyl β-D-1-thiogalactopyranoside (IPTG), Triton X-100, guanidine hydrochloride (GdnHCl), trifluoroacetic acid, D_2_O, 2,2-dimethyl-2-siapentane-5-sulfonate (DSS), and ampicillin were purchased from Sigma-Aldrich (St. Louis, MO, USA). ^15^N-labeled ammonium chloride was purchased from Cambridge Isotope Laboratories, Inc. (Tewksbury, MA, USA). Phenylmethylsulfonyl fluoride (PMSF) was purchased from Thermo Fisher Scientific (Waltham, MA, USA). Coomassie Brilliant Blue G-250 was purchased from J.T. Baker Chemical Co. (Phillipsburg, NJ, USA). Sodium azide was purchased from Merck (Millipore, Burlington, MA, USA).

### 2.2. Construction of the Expression Plasmid

**Figure 1:**
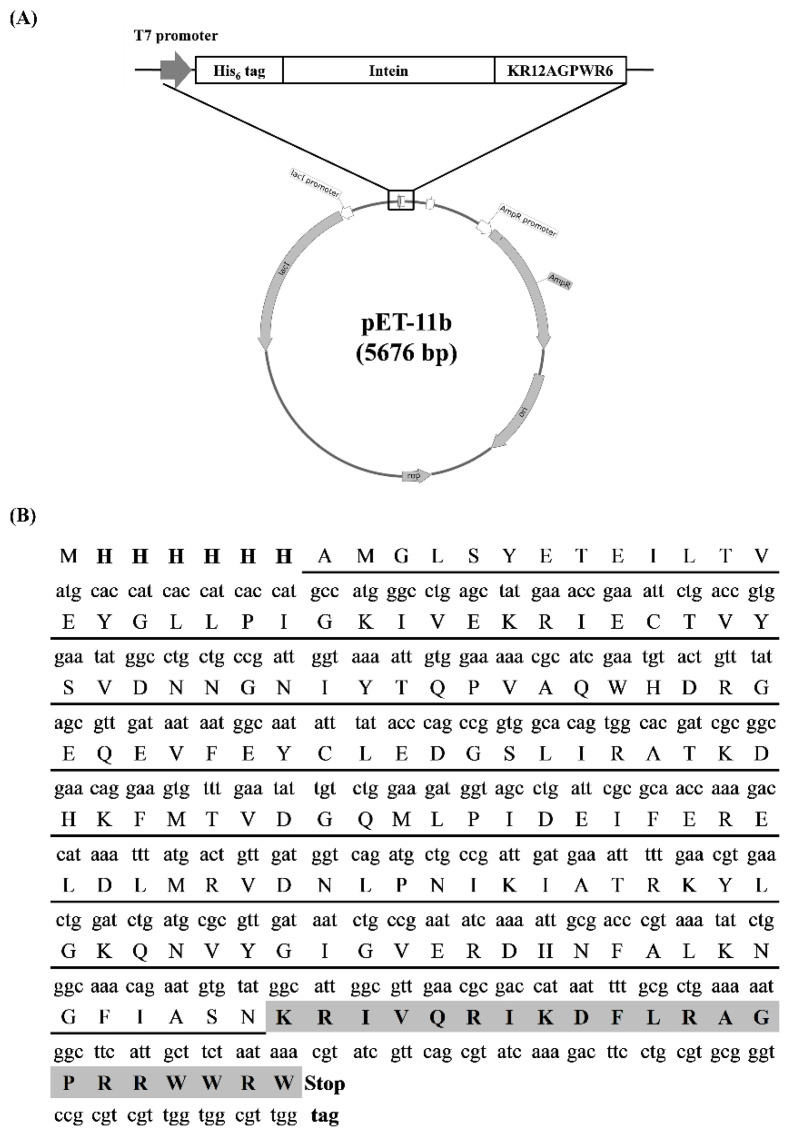
Gene map of the recombinant plasmid pET11b-His6-intein-KR12AGPWR6. (**A**) The illustration of inserted His6-intein-KR12AGPWR6 sequence. (**B**) Optimized DNA sequence and codon map. His6-tag are in bold; the sequence of intein is underlined; the sequence of KR12AGPWR6 is shaded.

Six histidines were attached in the N-terminus of intein, and the sequence of KR12AGPWR6 was attached to the C-terminus of intein (Figure 1A). The amino acid sequences and optimized DNA sequences are shown in Figure 1B. The pET11b-His6-intein construct was synthesized from Protech Technology Enterprise Co., Ltd. (Taipei, Taiwan).

### 2.3. Expression of His6-Intein-KR12AGPWR6

The constructed expression vector was inserted into *E. coli* BL21 (DE3) competent cells for expression. A single colony was selected and incubated in 100 mL LB broth containing ampicillin (0.1 mg/mL) at 37 °C with 150 rpm shaking overnight. A total of 25 mL incubated cell culture was then inoculated into 1000 mL LB broth containing ampicillin (0.1 mg/mL) until the OD_600_ reached 0.6 to 0.8. The protein was subsequently induced with 0.4 mM IPTG for 24 h at 20 °C. After induction, the cells were harvested by centrifugation at 6000× *g* for 20 min at 4 °C. The supernatant was discarded and the pellets were stocked at −20 °C.

### 2.4. Purification of His6-Intein-KR12AGPWR6

The cell pellets were resuspended in lysis buffer (20 mM sodium phosphate, 150 mM NaCl, 0.5% Triton X-100, 1 mM PMSF, pH 8.0) and lysed with a High-Pressure Homogenizer (AVESTIN EmulsiFlex C3, Mannheim, Germany). The cell lysates were separated with high-speed centrifugation at 4 °C (12,500× *g*, 30 min), and the supernatant was filtered with 0.2 µm filter before being applied to a Ni-NTA resin (QIAGEN, Hilden, Germany) equilibrated with lysis buffer. The target protein sample was separated under different concentrations of imidazole. The imidazole concentration was 40 mM at the washing steps and 400 mM at the elution steps, respectively. The eluted samples were concentrated using Amicon^®^ Stirred Cells with 3 kDa membrane (Merck Millipore, Burlington, MA, USA), and followed by dialysis using the cellulose tubular membrane (3.5 kDa MWCO, Cellu·Sep T1 Membrane, Membrane Filtration Products, Inc., Seguin, TX, USA) within 20 mM phosphate buffer (pH 8.0) at 4 °C for 24 h.

### 2.5. Intein Self-Cleavage

Self-cleavage of purified His6-Intein-KR12AGPWR6 was performed in high pH buffer (20 mM phosphate buffer, pH 10.0) at 55 °C for 72 h. After intein self-cleavage, the rKR12AGPWR6 was precipitated in the pellet. Then, the rKR12AGPWR6 peptide was obtained by high-speed centrifugation (12,500× *g*, 30 min, 4 °C), and the supernatant was discarded. The rKR12AGPWR6 peptide was further resuspended by 6 M guanidine hydrochloride before the purification by RP-HPLC [36].

### 2.6. Purification of rKR12AGPWR6 by RP-HPLC

All of the samples were purified by using C18 reversed-phase high-performance liquid chromatography (RP-HPLC) on the Prominence HPLC System (Shimadzu, Kyoto, Japan) and using a COSMOSIL C_18_-AR-II column (Nacalai Tesque, Kyoto, Japan). The column was equilibrated with ddH_2_O containing 0.1% (*v*/*v*) trifluoroacetic acid (TFA) and eluted with a gradient step from 15 to 100% (*v*/*v*) methanol containing 0.1% (*v*/*v*) TFA for 75 min at a flow rate of 1 mL/min. Signals were detected by UV 220 nm. The protein samples were collected and lyophilized, then resuspended in water, and the concentrations were determined by the bicinchoninic acid assay (BCA) method (GeneCopoeia^TM^, Rockville, MD, USA). The molecular mass of KR12AGPWR6 was verified by matrix-assisted laser desorption ionization-time of flight (MALDI-TOF) mass spectrometry.

### 2.7. SDS-PAGE Analysis

The samples were mixed with sample buffer (0.2 M Tris-HCl, pH 6.8, 30% glycerol, 10% sodium dodecyl sulfate (SDS), 10 mM DTT, 0.05% bromophenol blue). The samples were loaded to 12% (*wt*/*v*) SDS-polyacrylamide gel electrophoresis (SDS-PAGE) and the gel ran at 120 V for 80 min. Protein bands were detected by Coomassie Brilliant Blue G-250 staining.

### 2.8. Antimicrobial Activity Assays

*Staphylococcus aureus* ATCC 25923, *Escherichia coli* ATCC 25922, *Pseudomonas aeruginosa* ATCC 27853, and *Acinetobacter baumannii* 14B0100, were purchased from Bioresources Collection & Research Center (BCRC, FIRDI, Hsinchu, Taiwan). The antibacterial activities of peptides were determined by the standard broth microdilution method according to the guidelines of the Clinical and Laboratory Standards Institute (CLSI) [37]. Briefly, the bacteria were incubated in MHB overnight at 37 °C. The cell cultures were regrown to mid-log phase and subsequently diluted to a final concentration of 5 × 10^5^ CFU/mL. The peptides were loaded into each well at the final concentration of 64, 32, 16, 8, 4, 2, 1 μg/mL, then the microbes were loaded into each well of a polypropylene 96-well plate. After 16 h of incubation at 37 °C, the MIC values of peptides were determined by inspecting the visible growth of bacteria. The MIC values were defined as the lowest concentration of an antimicrobial agent that inhibits the visible growth of a microorganism. All experiments were repeated three times independently.

### 2.9. Circular Dichroism Spectroscopy

Circular dichroism spectra were recorded in the far-UV spectral region (190 to 260 nm) at 25 °C using a 0.1 cm path-length cuvette on an AVIV CD spectrometer (Aviv Biomedical Inc., Lakewood, NJ, USA). The peptide concentration was 60 µM in 20 mM sodium phosphate buffer or in 30% TFE buffer at pH 7.4.

### 2.10. Nuclear Magnetic Resonance Spectroscopy (NMR)

The NMR experiments were performed using 0.6 mM of ^15^N-labeled samples. The NMR samples were prepared in NMR buffer (20 mM sodium phosphate buffer, 10 mM NaN_3_, pH 4.5) with 10% D_2_O (*v*/*v*) for field/frequency lock. ^15^N-edited 2D HSQC (Heteronuclear Single-Quantum Correlation) spectroscopy; NOESY (Nuclear Overhauser Effect Spectroscopy); and TOCSY (Total Correlation Spectroscopy) experiments were recorded at 298 K on a Bruker Avance 600-MHz NMR spectrometer (Bruker, Billerica, MA, USA). ^1^H NMR data were referenced to ^1^H resonance frequency of DSS (2,2-dimethyl-2-siapentane-5-sulfonate). Quadrature detection in the indirect dimensions was determined by using the States-TPPI (time-proportional phase incrementation) method. Signals from H_2_O were suppressed through low power presaturation (pulse program: zgpr). An analysis of the spectra was conducted using the Sparky software (T.D. Goddard and D.G. Kneller, SPARKY3, University of California, San Francisco, CA, USA).

## 3. Results and Discussion

### 3.1. Construction of the Recombinant Plasmid

The pET-11b plasmid was used as a template and the designed construct His6-intein-KR12AGPWR6 was subcloned into the expression vector (Figure 1A). The N-terminal consecutive histidine served as purification tags. The amino acid sequence and the optimized DNA sequence were shown in Figure 1B. A DNA sequence analysis demonstrated that the His6-intein-KR12AGPWR6 sequence was correct.

### 3.2. Expression, Extraction and Purification of Recombinant His6-Intein-KR12AGPWR6

**Figure 2:**
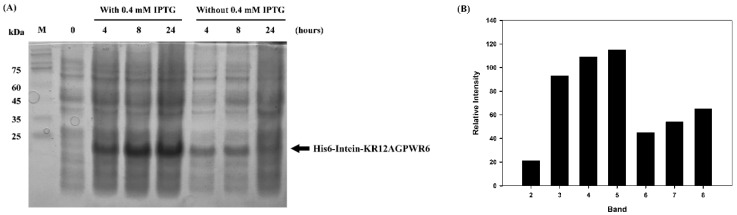
SDS-PAGE analysis of recombinant His6-Intein-KR12AGPWR6 in *E. coli* BL21. (**A**) SDS-PAGE of recombinant His6-Intein-KR12AGPWR6 expressed in *E. coli* BL21 with or without 0.4 mM IPTG at 20 °C for 4, 8, 24 h. Lane 1: protein molecular weight markers (kDa). Lane 2: before induction. Lanes 3–5: with IPTG induction at 20 °C for 4, 8, 24 h. Lanes 6–8: without IPTG induction at 20 °C for 4, 8, 24 h. (**B**) Relative intensities of the His6-Intein-KR12AGPWR6 bands. Proteins were visualized using Coomassie blue staining.

**Figure 3:**
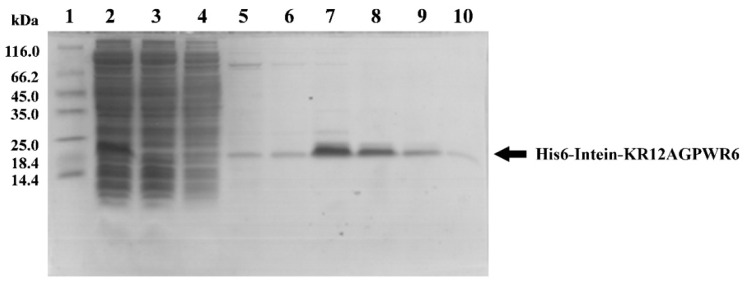
SDS-PAGE analysis of recombinant His6-Intein-KR12AGPWR6 expressed in *E. coli* at different steps of purification procedure. Lane 1: protein molecular weight markers (kDa); lane 2: supernatant of cell lysate; lane 3: flow through; lane 4–6: washing by 40 mM imidazole; lane 7–10: elution by 400 mM imidazole. Proteins were visualized using Coomassie blue staining.

*E. coli* BL21 (DE3) cells containing the pET11b-His6-intein-KR12AGPWR6 plasmid were successfully induced by IPTG, and the expression of His6-Intein-KR12AGPWR6 was analyzed by SDS-PAGE (Figure 2). As shown in Figure 2A, lanes 3 and 4 represented expression of His6-Intein-KR12AGPWR6 at 20 °C for 4, 8, and 24 h, respectively. The bands of His6-Intein-KR12AGPWR6 were observed, and the protein levels increased along with the induction time. As shown in Figure 2B, the relative intensity of lane 5 (24 h induction with IPTG) displayed the highest level of protein expression. The enhanced background impurities in lane 5 corresponding to 24 h induction with IPTG were removed by Ni-NTA purification (Figure 3, lane 4–6). Lanes 6 to 8 showed protein expression levels without IPTG induction and smaller amounts of recombinant proteins were observed (Figure 2A). Subsequently, the pellets were resuspended in lysis buffer and lysed with a high-pressure homogenizer. Ni-NTA resin was used for protein purification. As can be seen in Figure 3, the targeted proteins were examined in cell lysate (lane 2). No target proteins could be observed in the solution flow through (lane 3). The recombinant proteins were washed three times by 40 mM imidazole (lanes 4–6) to remove non-specific targets, and most of the non-specific targets were washed out the first time. Intein may perform its self-cleavage activity at low salt concentrations. In our previous study, we proposed a ‘prohibition condition’ to inhibit the self-cleavage activity of intein before using Ni-NTA column to purify the target protein [38]. The ‘prohibition condition’ in this intein system includes a condition with salt concentration > 300 mM and pH < 7. Therefore, we chose 400 mM imidazole to prevent intein self-cleavage before the purification steps. The target proteins were then eluted by 400 mM imidazole (lane 7–10) and the protein band of recombinant His6-Intein-KR12AGPWR6 (MW 19.7 kDa) was observed on SDS-PAGE at about 20 kDa (Figure 3). Imidazole was removed from the eluted sample using cellulose tubular membrane (3.5 kDa MWCO) in dialysis buffer at 4 °C for 24 h before intein self-cleavage.

### 3.3. Optimization of Intein’s Self-Cleavage

**Figure 4:**
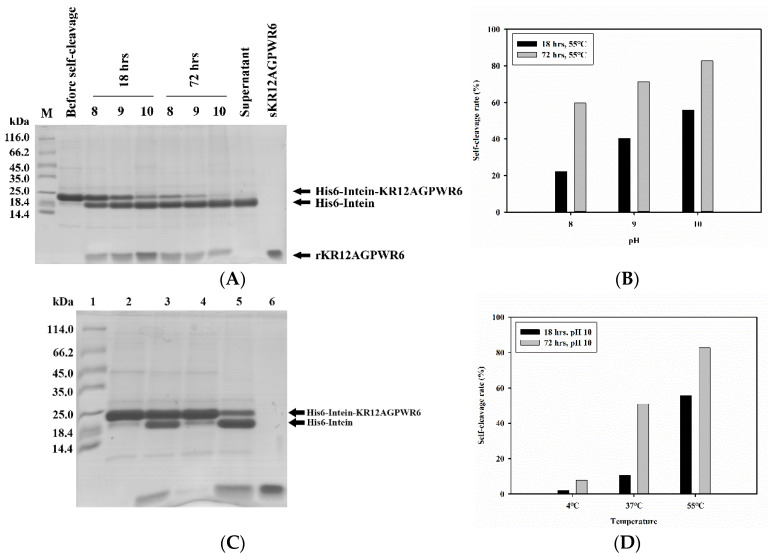
SDS-PAGE analysis of pH, temperature, and time optimization for intein’s self-cleavage. (**A**) SDS-PAGE analysis of pH optimization for intein’s self-cleavage. Lane 1: protein molecular weight markers (kDa); lane 2: protein in pH 10 buffer at 4 °C before self-cleavage; lanes 3–5: protein in different pH at 55 °C for 18 h; lanes 6–8: protein in different pH at 55 °C for 72 h; lane 9: the supernatant of protein in pH 10 at 55 °C for 72 h; lane 10: chemical synthesized sKR12AGPWR6 was used as a control. (**B**) Quantification of intein self-cleavage rates. (**C**) SDS-PAGE analysis of temperature optimization for intein’s self-cleavage. Lane 1: protein molecular weight markers (kDa). Lane 2 and 3: the targeted proteins were cleaved in pH 10 buffer for 18 h at 4 °C and 37 °C, respectively. Lane 4 and 5: targeted proteins were cleaved in pH 10 buffer for 72 h at 4 °C and 37 °C, respectively. Lane 6: synthetic KR12AGPWR6 was used as a control. (**D**) Quantification of intein self-cleavage rates. Proteins were observed using Coomassie blue staining.

Reaction time, pH, and temperature conditions were used to optimize intein’s self-cleavage. After intein’s self-cleavage, the mixtures were analyzed by SDS-PAGE (Figure 4A). Three major bands belonging to His6-intein-KR12AGPWR6, His6-intein, and KR12AGPWR6 were seen, and their molecular weights were 19.7 kDa, 16.9 kDa, and 2.8 kDa, respectively (Figure 4A, lane 3–8). During intein self-cleavage, we observed that KR12AGPWR6 precipitated in the pellet. Thus, it is difficult to use the quantification of KR12AGPWR6 on SDS-PAGE to find the optimized intein self-cleavage condition. On the other hand, His-intein-KR12AGPWR6 and His6-intein dissolved in the supernatant. Therefore, we used the quantification of His-intein-KR12AGPWR6 and His6-intein on SDS-PAGE to determine the best self-cleavage conditions of intein. The band of KR12AGPWR6 was the same as synthetic KR12AGPWR6 (MW 2.8 kDa) (lane 10), which suggested that KR12AGPWR6 was released via intein’s self-cleavage. To optimize intein’s self-cleavage efficacy, purified His6-intein-KR12AGPWR6 was kept in various pH buffers at 55 °C for 18 and 72 h. As shown in Figure 4A,B, the self-cleavage rate of intein at 72 h was higher than 18 h under different pH conditions. Further, a higher pH (pH = 10) exhibited better intein self-cleavage activity. Similarly, intein self-cleavage activity at 72 h was higher than 18 h under different temperatures. The best intein cleavage condition for His6-Intein-KR12AGPWR6 was pH 10, 55 °C, for 72 h. Moreover, intein lost its self-cleavage activity at 4 °C, while the self-cleavage activity recovered at 37 °C. These results suggested that the most optimized conditions for the self-cleavage of His6-intein-KR12AGPWR6 were under high pH, high temperature, and longer reaction time.

### 3.4. Purification of KR12AGPWR6

**Figure 5:**
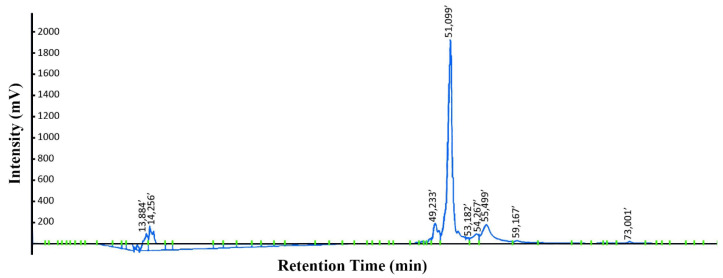
Reversed-phase HPLC purification of KR12AGPWR6 after intein’s self-cleavage. The samples were resuspended with 6 M guanidine hydrochloride and purified by reversed-phase HPLC on the Prominence HPLC System equipped with a C18 column. The column was equilibrated with ddH_2_O containing 0.1% (*v*/*v*) trifluoroacetic acid (TFA) and the gradient ranging from 15 to 100% (*v*/*v*) methanol containing 0.1% (*v*/*v*) TFA for 75 min at a flow rate of 1 mL/min. Signals were detected by UV 280 nm.

**Figure 6:**
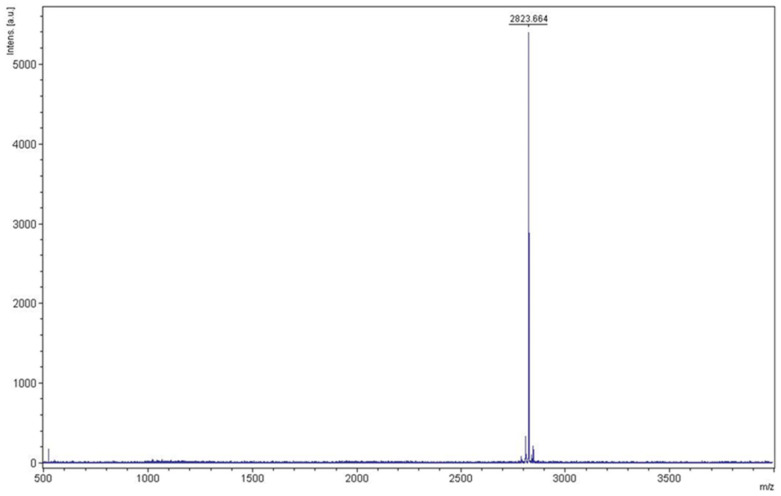
Mass analysis of KR12AGPWR6 from RP-HPLC. The molecular weight of the purified KR12AGPWR6 was found to be 2823.664 Da. The theoretical MW of KR12AGPWR6 was calculated to be 2824.3 Da.

KR12AGPWR6 was redissolved by using 6 M guanidine hydrochloride before RP-HPLC purification. Reversed-phase HPLC was used to purify KR12AGPWR6 with a gradient of water/methanol containing 0.1% trifluoroacetic acid (TFA). The prolife of HPLC chromatograms is displayed in Figure 5. The retention time of KR12AGPWR6 was found at 51 min. The yield of KR12AGPWR6 was 2.41 ± 0.33 mg/L. The molecular weight of the eluted KR12AGPWR6 was determined to be 2823.664 Da by MALDI-TOF MS (Figure 6), which was close to the theoretical MW (2824.3 Da).

### 3.5. Antimicrobial Activity

The antimicrobial activities of the recombinant rKR12AGPWR6 and chemically synthesized sKR12AGPWR6 against *S. aureus* ATCC 25923, *E. coli* ATCC 25922, *P. aeruginosa* ATCC 27853, and *A. baumannii* BCRC 14B0100 were evaluated by MIC assay. As shown in Table 1, the MIC value of rKR12AGPWR6 was the same as chemically synthesized sKR12AGPWR6 against *S. aureus* ATCC 25,923 (2 µg/mL) and *A. baumannii* BCRC 14B0100 (1 µg/mL). The recombinant rKR12AGPWR6 had a MIC of 4 µg/mL against *E. coli* ATCC25922 and *P. aeruginosa* ATCC 27853. The results showed that rKR12AGPWR6 exhibited similar antimicrobial activities as the chemically synthesized sKR12AGPWR6 against both Gram-positive and Gram-negative bacteria.

### 3.6. Characterization of the Recombinant rKR12AGPWR6 by CD and NMR

**Figure 7:**
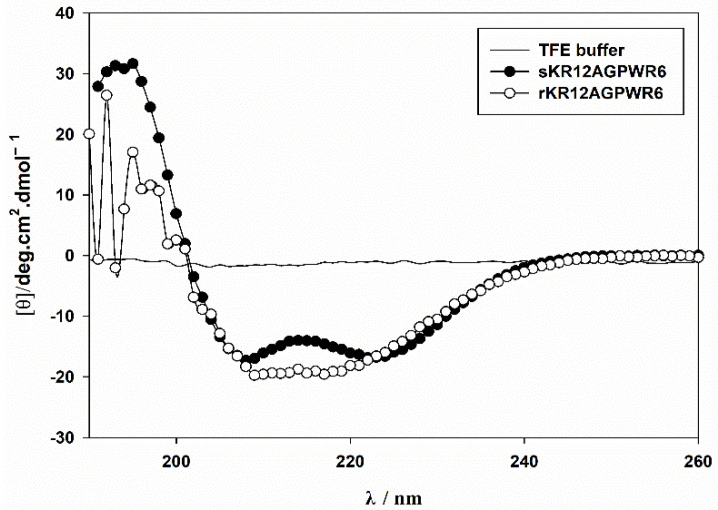
Circular dichroism spectra of synthetic and recombinant KR12AGPWR6 in different environments. Circular dichroism spectra of 60 µM synthetic and recombinant KR12AGPWR6 in 30% TFE buffer in pH7.4 at 25 °C.

**Figure 8:**
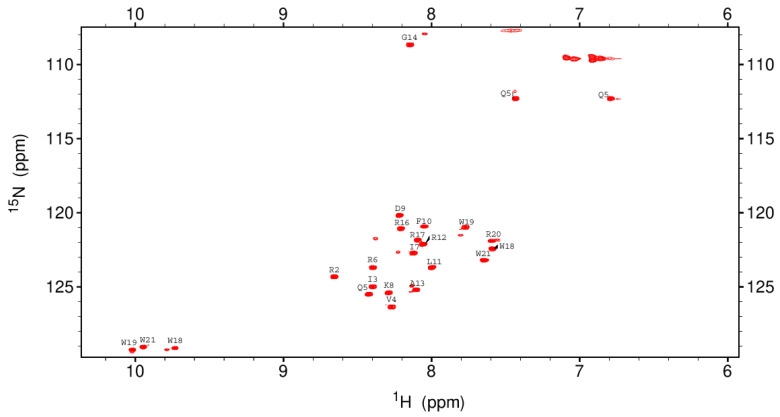
^1^H-^15^N HSQC spectra of 0.6 mM rKR12AGPWR6 in 20 mM sodium phosphate buffer, pH 4.5, 298 K. The ^15^N-labeled samples were expressed in *E. coli* BL21 (DE3) cells grown in M9 medium containing ^15^N-labeled ammonium chloride (1 g/L), and the purification of peptides as mentioned above. The cross peaks of rKR12AGPWR6 were shown in red.

CD spectroscopy was used to compare the structures of the recombinant rKR12AGPWR6 and the chemically synthesized sKR12AGPWR6 (Figure 7). The CD results indicated that both the recombinant and chemically synthesized KR12AGPWR6 adopted a typical α-helical structure under 30% TFE buffer. In order to achieve backbone assignments of rKR12AGPWR6, the 2D TOCSY and NOESY spectra of KR12AGPWR6 in 20 mM phosphate buffer were obtained (Figure S1). In addition, we successfully assigned the ^1^H and ^15^N backbone resonance peaks of rKR12AGPWR6 in buffer. A well-resolved ^1^H-^15^N HSQC spectrum of the fingerprint region of rKR12AGPWR6 is shown in Figure 8.

### 3.7. Design of New Cecropin-like Peptides

Cecropin analogues [38], Cecropin A/Cecropin B hybrids [39], and Cecropin A, LL-37, and Magainin hybrids exhibited strong antimicrobial and anticancer activities [40]. Many other AMPs, such as chicken cathelicidin fowlicidin-2, MSI-594, SMAP-29, Pardaxin, Cecropin A, and Papiliocin, with similar Cecropin-like structural features also possessed both antimicrobial and LPS neutralizing abilities [19,41,42,43,44,45].

Recently, Malmsten and co-workers developed a strategy to increase the salt resistance of short antimicrobial peptides by adding tryptophan and/or phenylalanine end-tags [46,47,48]. End-tagging was also found to promote other biological effects, such as anti-cancer and receptor binding activities [49,50]. We modified this strategy by adding β-naphthylalanine (Nal) to the termini of the short antimicrobial peptide S1 (Ac-KKWRKWLAKK-NH_2_) to boost its salt resistance, serum proteolytic stability, and antiendotoxin activities [51,52]. We used solution NMR and paramagnetic relaxation enhancement techniques to study the structural differences of S1 and S1-Nal-Nal in LPS micelles [53]. The three-dimensional structures of S1 and S1-Nal-Nal in complex with LPS clearly provided an explanation for the differences in their antiendotoxin activities. Based on these structural results and the above-mentioned anti-LPS AMPs, we proposed a possible model to explain the mechanism of S1-Nal-Nal in the interaction with LPS. Firstly, S1-Nal-Nal adopts a random coil structure in aqueous solution. Then, it is attracted to LPS by the electrostatic interactions between the induced amphipathic helix and the negatively charged region of LPS. Finally, the bulky hydrophobic β-naphthylalanine (Nal) end-tags insert themselves into LPS by extra hydrophobic interactions with the lipid A region of LPS. The LPS-induced inflammation is then prohibited by the blocked lipid A region.

Based on the above-mentioned structural and functional studies of Cecropins and S1-Nal-Nal, we suspect that the binding and neutralization of LPS is not just through the sequences of Cecropins because some of the Cecropins and Cecropin analogues, although different in their sequences, can still bind to and neutralize LPS induced pro-inflammatory effects [18,40,46]. Therefore, we hypothesize that the binding and neutralization of LPS occurs through their specific structural features (i.e., amphipathic helix-AGP hinge-hydrophobic helix). Herein, we propose to extend this strategy to design AMPs with enhanced salt resistance and antiendotoxin activity. Some of the potential AMPs are listed in Table 2.

### 3.8. Expression and Purification of Cecropin-like AMPs

Intein is a protein segment that can cleave itself from a whole protein sequence and ligate the remaining N-terminal and C-terminal portions (the exteins) with a peptide bond [21]. Until now, over 600 inteins with different lengths have been identified. The specific cleavage-ligation function of intein has enabled various applications, such as protein engineering, isotope labeling, biomaterials, cyclization, and protein purification [54,55]. Recently, we have created an N-terminal mutated intein that has no N-terminal cleavage activity but preserves its C-terminal cleavage activity [21]. We have shown that this mutated intein can be used as a fusion tag and can self-cleave from the target protein. Moreover, we have shown that the efficiency of the intein self-cleavage is dependent on ionic strength, pH, temperature, and reaction time [21].

In this study, we used the mutated intein as a fusion partner for the expression and purification of KR12AGPWR6. We successfully expressed His6-intein-KR12AGPWR6 in *E. coli* (Figure 2) and the recombinant His6-intein-KR12AGPWR6 was purified by Ni-NTA column (Figure 3). In order to optimize the self-cleavage efficacy of His6-intein-KR12AGPWR6, the recombinant proteins were incubated at 55 °C under various pH buffers. The optimal pH for intein self-cleavage was 10. As can be seen in Table 1, the recombinant rKR12AGPWR6 possessed strong antimicrobial activities against both Gram-positive and Gram-negative bacteria, similar to the activities of the synthesized sKR12AGPWR6. We have also shown that the expressed rKR12AGPWR6 adopts an α-helical structure in TFE which is identical to the chemical synthesized sKR12AGPWR6. In addition, we demonstrated that the ^15^N-labeled rKR12AGPWR6 that was produced in this study may be used to understand the structural features and interactions between rKR12AGPWR6 and model membranes/microbes for the design and development of useful antimicrobial peptides.

The lower yield of KR12AGPWR6 could be due to its antimicrobial activity, which makes itself potentially fatal to the expression host. However, the yield of KR12AGPWR6 is comparable to other antimicrobial peptides expressed by using the thioredoxin fusion or the GST fusion protein systems [56]. The premature intein self-cleavage before Ni-NTA column purification is also an intrinsic problem associated with the intein system. This problem causes loss in the yield of the target peptide. On the other hand, we could easily obtain the purified product by RP-HPLC by using this intein self-cleavage system. This intein self-cleavage system also requires no auxiliary enzymes or chemicals to remove the carrier protein. Additionally, by using bioreactors, volumetric protein production (*E. coli* cell density) could be improved up to 10–34 fold using a fed-batch strategy compared to batch cultivation [57]. Therefore, this intein self-cleavage system presents a potential method to produce AMPs in *E. coli* and is beneficial to reducing the cost of production for commercial scale production.

In addition to KR12AGPWR6, we chose CecropinA-AGP-WR6 (KWKLFKKIEKVGQNIRDGIIK-**AGP**-RRWWRW) (Table 2) to test this intein self-cleavage system. Figures S2 and S3 demonstrated that CecropinA-AGP-WR6 can be successfully obtained by using this intein self-cleavage expression and purification system. As shown in Table 3, CecropinA-AGP-WR6 also possesses strong antimicrobial activities against Gram-negative and Gram-positive bacteria.

In conclusion, we successfully expressed the cecropin-like peptides rKR12AGPWR6 and CecropinA-AGP-WR6 from *E. coli*, and purification of rKR12AGPWR6 and CecropinA-AGP-WR6 was efficiently carried out by using the optimized intein self-cleavage system. This study provided a potential strategy to produce recombinant AMPs up to a commercial scale production, and this intein self-cleavage system can be widely applied to obtain other AMPs from *E coli*.

## Figures and Tables

**Table 1:** MICs of sKR12AGPWR6 and rKR12AGPWR6 against Gram-positive and Gram-negative bacteria.

Bacterial Strains	Minimum Inhibitory Concentration (μg/mL)	
	sKR12AGPWR6	rKR12AGPWR6
*S. aureus* ATCC 25923	2	2
*E. coli* ATCC 25922	2	4
*P. aeruginosa* ATCC 27853	2	4
*A. baumannii* BCRC 14B0100	1	1

**Table 2:** Proposed AMPs based on amphipathic helix-AGP-hydrophobic helix.

Magainin-AGP-WR6	GIGKFLHSAKKFGKAFVGEIMNS-**AGP**-RRWWRW
KR12-AGP-Cecropin P1	KRIVQRIKDFLR-**AGP**-IAIAIQGGPR
Cecropin P1-AGP-WR6	SWLSKTAKKLENSAKKRISE-**AGP**-RRWWRW
KR12-AGP-Cecropin A	KRIVQRIKDFLR-**AGP**-AVAVVGQATQIAK
CecropinA-AGP-WR6	KWKLFKKIEKVGQNIRDGIIK-**AGP**-RRWWRW
Melittin-AGP-WR6	GIGAVLKVLTTGLPALISWIKRKRQQ-**AGP**-RRWWRW
Melittin-AGP-Cecropin P1	GIGAVLKVLTTGLPALISWIKRKRQQ-**AGP**-IAIAIQGGPR

**Table 3:** MIC values of recombinant CecropinA-AGP-WR6 against Gram-positive and Gram-negative bacteria.

Bacterial Strains	Minimum Inhibitory Concentration (μg/mL)
*S. aureus* ATCC 29213	16
*E. coli* ATCC 25922	8
*P. aeruginosa* ATCC 27853	8
*A. baumannii* BCRC 14B0100	2

## Data Availability

The data presented in this study are available on request from the corresponding author.

## Supplementary Materials

The following supporting information can be downloaded at: https://www.mdpi.com/article/10.3390/biomedicines10061351/s1, Figure S1: Backbone assignment of KR12AGPWR6.; Figure S2: SDS-PAGE analysis of recombinant His6-Intein-CA-AGP-WR6 expressed in *E. coli*.; Figure S3: Mass analysis of CA-AGP-WR6 from RP-HPLC.
