# Supplementary material for: High Level Expression and Purification of Cecropin-like Antimicrobial Peptides in Escherichia coli
Source: Biomedicines. 2022 Jun 8;10(6):1351. doi: 10.3390/biomedicines10061351 (PMC9220022; doi:10.3390/biomedicines10061351)
Supplement: Supplementary file 1 [file biomedicines-10-01351-s001.zip › biomedicines-1754810-supplementary.pdf]

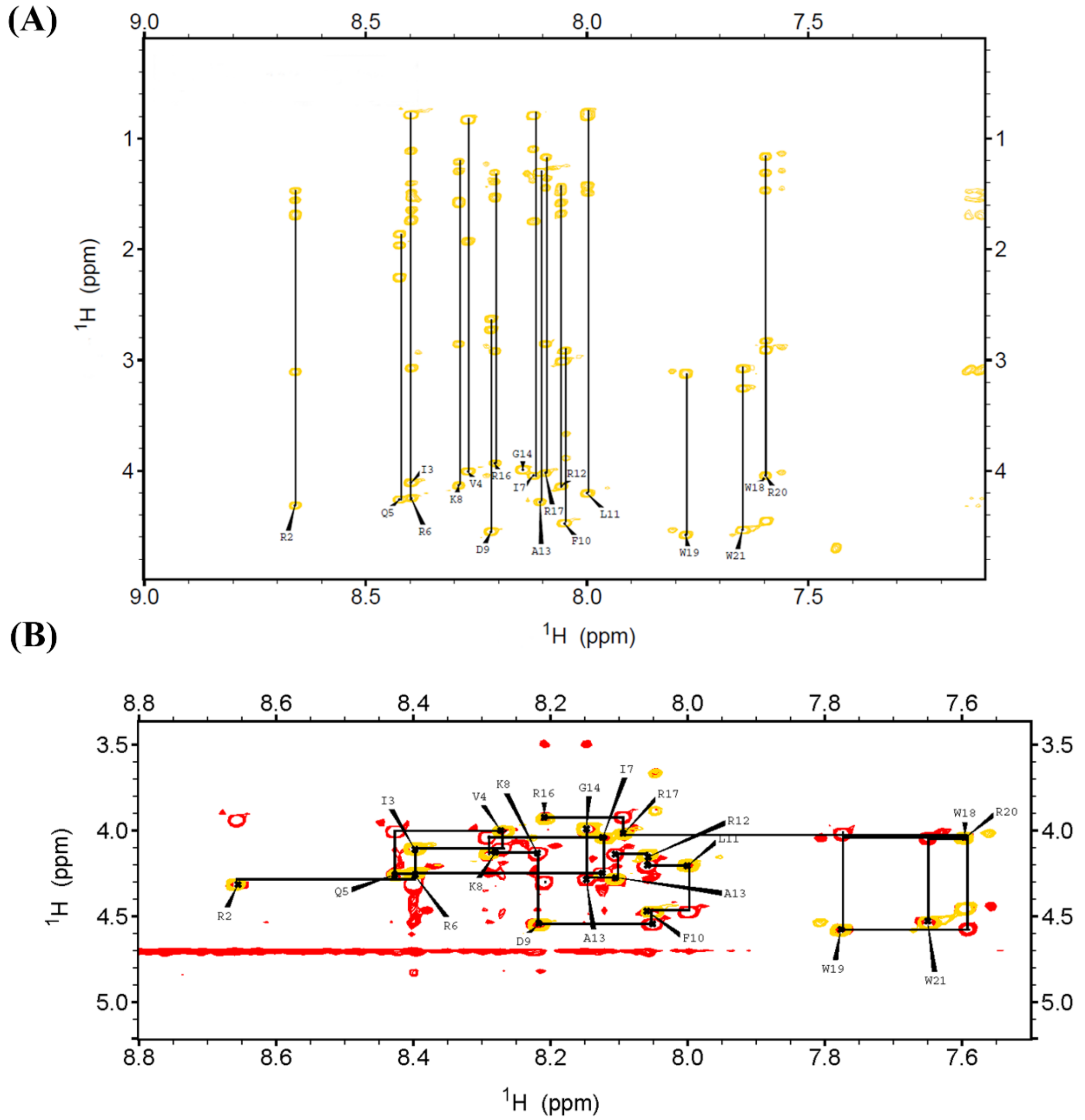

Figure S1. Backbone assignment of KR12AGPWR6. (**A**) 600 MHz TOCSY spectra assignments of KR12AGPWR6. (**B**) Overlapped spectra of TOCSY (yellow) and NOESY spectra (red) at NH-CαH region for KR12AGPWR6.

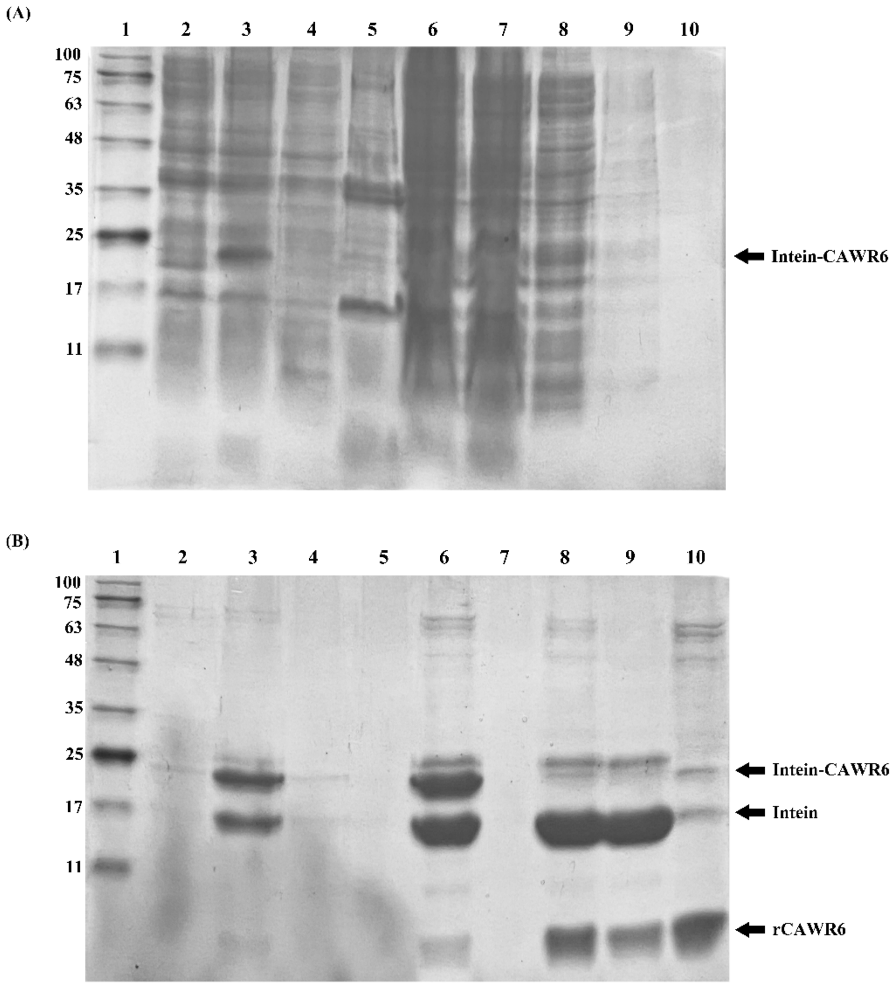

Figure S2. SDS-PAGE analysis of recombinant His6-Intein-CA-AGP-WR6 expressed in *E. coli*. (**A**) Lane 1: protein markers (kDa); lane 2: pre-culture cell lysate; lane 3: after IPTG induction at 16°C for 24 hours; lane 4: before IPTG induction; lane 5: cell lysate after IPTG induction at 16°C for 24 hours; lane 6: supernatant of cell lysate; lane 7: flow through; lane 8-10: washing by 40 mM imidazole. (**B**) Lane 1: protein markers (kDa); lane 2: washing by 80 mM imidazole; lane 3-5: elution by 400 mM imidazole; lane 6: eluted proteins after concentration using Amicon stirred cells with 3 kDa membranes; lane 7: flow through from lane 6; lane 8: mixtures of samples after intein self-cleavage in pH 10 buffer at 37°C for 24 hours; lane 9: supernatant of proteins after intein self-cleavage in pH 10 at 55°C for 24 hours; lane 10: pellet of proteins after intein self-cleavage in pH 10 at 55°C for 24 hours. Proteins were visualized with Coomassie blue staining.

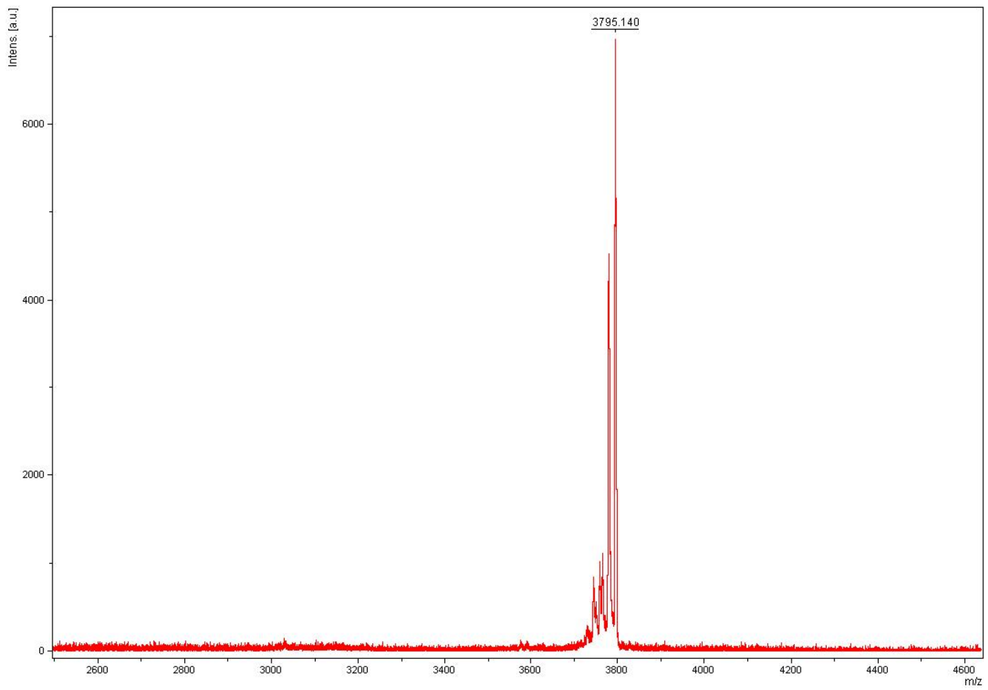

Figure S3. Mass analysis of CA-AGP-WR6 from RP-HPLC. Molecular weight of CA-AGP-WR6 was found to be 3795.140 Da based on MALDI-TOF Mass. The theoretical MW of CA-AGP-WR6 was calculated to be 3794 Da.
